# Seasonal Incidence of Medically Attended Respiratory Syncytial Virus Infection in a Community Cohort of Adults ≥50 Years Old

**DOI:** 10.1371/journal.pone.0102586

**Published:** 2014-07-15

**Authors:** David L. McClure, Burney A. Kieke, Maria E. Sundaram, Melissa D. Simpson, Jennifer K. Meece, Frangiscos Sifakis, Robert A. Gasser, Edward A. Belongia

**Affiliations:** 1 Marshfield Clinic Research Foundation, Marshfield, Wisconsin, United States of America; 2 MedImmune LLC, Gaithersburg, Maryland, United States of America; University of Tennessee Health Science Center, United States of America

## Abstract

**Background:**

Diagnostic testing for respiratory syncytial virus (RSV) is not routinely performed in adults. We estimated medically attended RSV seasonal incidence in a community cohort of adults ≥50 years old during four influenza seasons (2006–07 through 2009–10).

**Methods:**

Patients seeking care for acute respiratory illness (ARI) were prospectively enrolled and tested for RSV by multiplex RT-PCR. Results from enrolled patients were used to estimate projected cases among non-enrolled patients with ARI. The seasonal incidence of medically attended RSV was the sum of actual and projected cases divided by the community cohort denominator. Since each enrollment period did not include the entire RSV season, incidence estimates were adjusted to account for the statewide proportion of RSV occurring outside the study enrollment period.

**Results:**

There were 16,088 to 17,694 adults in the cohort each season and 164 RSV cases in all 4 seasons. The overall seasonal incidence of medically attended RSV was 154 episodes (95% CI, 132–180) per 10,000 persons; the incidence was highest in 2007–08 (179) and lowest in 2006–07 (110). Among persons 50–59, 60–69, and ≥70 years old, RSV incidence was 124 (95% CI, 99–156), 147 (95% CI, 110–196), and 199 (95% CI, 153–258), respectively.

**Conclusions:**

The incidence of medically attended RSV increased with age and was similar during four seasons.

## Introduction

Respiratory syncytial virus (RSV) has been increasingly recognized as a cause of serious respiratory illness in adults. Most studies of RSV infection in adults have focused on hospitalized patients, and risk factors for RSV-related hospital admission include chronic pulmonary disease, functional disability, low neutralizing antibody titer, and immunocompromised status [1], [2]. RSV is also an important cause of community-acquired pneumonia in adults [3]. RSV infections typically follow a seasonal pattern that overlaps periods of influenza circulation, although the epidemic peaks often differ [4].

The incidence and burden of illness caused by RSV has not been clearly established in adult populations. Clinical recognition is limited because the symptoms of RSV illness are similar to those of other respiratory viruses, and highly sensitive diagnostic tests for RSV are not routinely performed. Furthermore RSV diagnostic tests, especially rapid-antigen tests, are less sensitive in adults compared to children because of much lower levels of virus that are shed [5]. A surveillance study identified 211 RSV infections in healthy adults from 1975 through 1995; 84% were symptomatic, and one quarter of the symptomatic infections involved the lower respiratory tract [6]. A prospective cohort study involving 608 healthy older adults over 4 winter seasons (2000–2003) reported an annual RSV incidence of 3% to 7% in the cohort; RSV generated fewer office visits than influenza [7]. Other studies [8]–[10] have assessed the presence of RSV in samples collected from patients with acute respiratory illness in emergency or inpatient settings. In contrast, data characterizing the population incidence and burden of RSV illness among older adults are scarce.

The current study was undertaken to estimate the seasonal incidence of medically attended RSV infection in a community cohort of adults ≥50 years old during four winter seasons.

## Methods

### Ethics Statement

The Marshfield Clinic Institutional Review Board (IRB) reviewed and approved this study. All participants gave verbal informed consent and consent was documented in study databases. Since the research presented no more than minimal risk and involved no procedures for which written consent was normally required outside of the research context, the verbal consent procedure was approved by the Marshfield Clinic IRB.

### Setting and participants

Marshfield Clinic Research Foundation (MCRF) has conducted influenza vaccine effectiveness studies funded by the Centers for Disease Control and Prevention (CDC) since 2004 [11]–[14]. Each season, patients with medically attended acute respiratory illness (MAARI) were enrolled from the population of community dwelling individuals living in the central Marshfield Epidemiologic Study Area (MESA). MESA is a dynamic, population-based cohort of approximately 54,000 individuals living in 14 zip codes surrounding Marshfield, Wisconsin. Nearly all MESA residents receive their inpatient and outpatient care from Marshfield Clinic facilities, which use an electronic medical record that captures 90% of outpatient visits, 95% of hospital discharges, and 99% of deaths for the population [15]. The analysis of RSV incidence was restricted to the 2006–07 through 2009–10 seasons when all adults ≥50 years old were recruited if they met symptom eligibility criteria.

Patients were actively screened and recruited by research staff during or after an outpatient or inpatient encounter for acute respiratory illness. Eligibility criteria were described previously [17]. Briefly, patients were eligible for study enrollment if they presented with symptoms of fever/feverishness, chills, or cough and had not initiated antiviral therapy. Potential participants with illness duration >7 days (>10 days in 2006–07) were excluded to minimize false negative test results. Research coordinators used an electronic appointment system to screen chief complaints and identify potential participants in primary care departments at the Marshfield Clinic main campus and a satellite clinic. Eligible patients were also recruited from the Emergency Department and at the time of admission to the only acute care hospital serving the study population (St. Joseph's Hospital). Many patients who were not approached during the clinical encounter were contacted by phone on the following day if they received an *International Classification of Diseases, Version 9, Clinical Modification* (ICD-9) diagnosis code indicating acute respiratory illness (codes available on request). Those who met eligibility criteria were invited to participate.

Each adult participant was interviewed at the time of enrollment to determine illness onset date and symptoms. Nasopharyngeal swabs were obtained and placed in M4-RT viral transport media for RT-PCR testing. After influenza testing was complete, sample aliquots were stored at −70°C until the current study was initiated to identify other respiratory viruses.

Seasonal enrollment start dates and duration were: January 22, 2007 (10 weeks), January 21, 2008 (10 weeks), and January 19, 2009 (12 weeks). In response to the 2009 pandemic, enrollment began on November 30, 2009 and continued for 26 weeks.

### Laboratory

Sample aliquots were stored at −70°C after initial influenza testing was completed for the vaccine effectiveness studies. Archived samples were tested for the presence of respiratory virus nucleic acid using a multiplex respiratory virus panel (eSensor Respiratory Viral Panel, GenMark Diagnostics, Inc., Carlsbad, CA). This multiplex panel tested for respiratory syncytial virus (RSV) A and B, human rhinovirus, human metapneumovirus, parainfluenza viruses 1–4, influenza A and B (including subtypes of influenza A), coronaviruses OC43, NL63, HKU1 and 229E, and adenoviruses B and E. Nucleic acid was extracted from the swabs using the Roche MagnaPure 2.0 system and was then amplified using RT-PCR with target-specific primers. Target-specific signals were determined by voltammetry, a process which generates electrical signals from ferrocene-labeled signal probes. We validated the GenMark multiplex assay RSV A and B against singleplex assays developed by CDC. The sensitivity and specificity was 98% and 99% for RSV (unpublished data), which was consistent with GenMark multiplex RT-PCR compared to singleplex in pediatric samples [16]. Multiplex RT-PCR results have been reported separately in adults for other viruses [17], and only RSV results were used for the current analysis.

### Seasonal Incidence Estimates

The seasonal incidence of RSV was defined as the number of medically attended RSV cases per 10,000 community residents ≥50 years old. We generated separate estimates by season, gender, and RSV type (A and B). In three seasons, study enrollment was limited to 10–12 weeks of widespread influenza activity. However, we standardized all seasonal incidence estimates to the time period from week 40 (early October) through week 18 (end of April). We used Wisconsin State Laboratory of Hygiene RSV surveillance data [18] to weight our incidence estimates for the shorter enrollment period to a standard 31 week time period each season (week 40 through week 18). This adjustment factor was based on the inverse of the proportion of all state-wide cases that occurred during the seasonal enrollment periods. For the 2009–10 season, enrollments were truncated at 2010 week 18 (enrollment duration 23 weeks) for this analysis.

The incidence calculations were based on two assumptions. First, we assumed that RSV test results from enrolled patients can be extrapolated to non-enrolled patients in the community cohort who had medically attended acute respiratory illness (MAARI) during the study enrollment period. We used electronic ICD-9 diagnosis codes for acute respiratory infections (i.e. MAARI codes) to identify these patients (specific list available on request). For each person in the cohort, respiratory illness visits were classified as either absent (0 visits) or present (≥1 visit) based on the presence or absence of a relevant MAARI code during the study enrollment period. The number of RSV episodes among nonenrolled cohort members was estimated by multiplying the proportion RSV positive (among enrolled individuals) by the number of nonenrolled individuals with a MAARI episode (defined by ICD-9 codes). Although rare, a few enrolled patients did not receive a diagnosis code for acute respiratory illness because eligibility was based on presenting symptoms rather than diagnosis codes. These individuals were included in the numerator if the RSV result was positive.

The second assumption was that the number of cases occurring outside the enrollment period in our cohort of older adults was proportional to the number of laboratory confirmed RSV cases in Wisconsin occurring outside the enrollment period. Wisconsin RSV cases were determined for the period week 40 through week 18, and for the specific study enrollment window each season using weekly data provided by the Wisconsin State Laboratory of Hygiene. The State Laboratory of Hygiene does not report cases by age group, but presumably the majority of laboratory confirmed infections were identified in children since clinical testing in adults is uncommon.

Poisson regression with analytic weights, offsets and robust variance estimation [19], [20] was used to estimate seasonal cumulative incidence with corresponding 95% confidence intervals, and to perform statistical tests comparing subgroups. The outcome in the Poisson models was the dichotomous virus test result (coded 1 = positive, 0 = negative). We calculated the numerator as the sum of enrolled participants with RT-PCR confirmed RSV and the estimated number of non-enrolled cohort members with RSV based on the assumptions listed above. Analytic weights were created by extrapolating RSV positivity proportions for enrolled patients with MAARI episodes to other nonenrolled cohort members with MAARI episodes within strata defined by age group (50–59, 60–69, and ≥70 years), gender and season. Enrolled patients with no MAARI episodes were assigned analytic weights of 1.0. The analytic weights were then multiplied by the appropriate state level adjustment factors as described above. The offset terms were stratified by season, age group and gender. Within each stratum, the offset was the natural logarithm of the following quantity: number of cohort members divided by the sum of analytic weights. All calculations were performed using SAS version 9.2 (SAS Institute, Cary, NC).

## Results

The cohort of eligible adults ≥50 years old ranged from 16,088 persons in 2006–07 to 17,694 in 2009–10. There were 20,453 unique individuals who were included in the cohort for at least 1 of the 4 seasons. Of these 13,355 (65%) were in all 4 seasons; 2,452 (12%) were in 3 seasons, and 2,313 (11%) were in two seasons. The mean age (±SD) of community cohort members was 64 years (±12) at the beginning of each season and 53% were female (Table 1). The age, gender, congestive heart failure (CHF) and chronic obstructive pulmonary disease (COPD) distributions were similar between the cohort of eligible adults and enrolled RSV infected patients. The percent of community cohort members who had a MAARI episode during the enrollment period (including those not enrolled) ranged from 6% to 12% (Table 2). There were 1,326 eligible enrollments across all seasons for adults ≥50 years old, and 164 (12%) were positive for RSV. Clinical characteristics of the RSV infections have been previously reported [17]; 93% presented with cough, 79% with nasal congestion, 65% with fever, and 51% with wheezing. Among the 164 RSV positive cases, 154 (94%) received at least one MAARI diagnosis code; the remaining 10 patients had a chief complaint of acute respiratory illness but did not receive a MAARI diagnosis code. The mean and median age of RSV infected patients was 64.2 and 61.5 years, and 101 (62%) were female (Table 1). The median interval from symptom onset to enrollment was 4 days for both for those testing positive and those testing negative for RSV. Seven (4%) of 164 RSV cases had preexisting CHF and 7 (4%) had COPD; 14 (9%) had RT-PCR evidence of infection with another virus in addition to RSV. Ten (6%) of 164 patients with RSV were hospitalized within 14 days after illness onset.

**Table 1:** Demographics of community cohort of adults ≥50 years old and RSV infected patients for all seasons combined (2006–07 to 2009–10).

	RSV Cases	Community Cohort
	(n = 164)	(n = 20,453)^1^
Age at enrollment in years (mean ± sd) [median]	64.2 (10.6)[61.5]	63.7 (12.1) [60.3]
Age group: N (%)		
50–59 years old	69 (42.0)	9955 (48.7)
60–69 years old	44 (26.9)	4475 (21.9)
≥70 years old	51 (31.1)	6023 (29.4)
Female: N(%)	101 (61.6)	10834 (53.0)
Comorbid conditions		
COPD^2^	7 (4.3)	1290 (6.3)
CHF^3^	7 (4.3)	596 (2.9)

1 There were 20,453 unique individuals who were included in the cohort for at least 1 of the 4 seasons. Of these 13,355 (65%) were in all 4 seasons; 2452(12%) were in 3 seasons, and 2313(11%) were in two seasons.

2 Chronic obstructive pulmonary disease (COPD) defined having an ICD-9 code within the range of 491.001–492.999 in the years 2005 to 2010 for cohort or prior to the study season for RSV cases.

3 Congestive heart failure (CHF) defined as having an ICD-9 code within the range of 428.001–428.9 in the years 2005 to 2010 for cohort or prior to the study season for RSV cases.

**Table 2:** Visits for acute respiratory illness, enrollments and RSV test results in a community cohort of adults ≥50 years old.

Season	2006–07	2007–08	2008–09	2009–10
Study enrollment period (weeks)	10	10	12	23
Percent of state RSV cases (all ages) occurring during enrollment period	63%	56%	72%	99%
No. persons in community cohort	16088	16881	17072	17694
No. persons with MAARI episode (based on ICD-9 codes) during enrollment period	901 (6%)	1332 (8%)	1269 (7%)	2133 (12%)
No. persons enrolled and tested	267	336	307	416
No. (%) positive for RSV among enrolled persons	26 (10%)	40 (12%)	46 (15%)	52 (13%)

**Figure 1:**
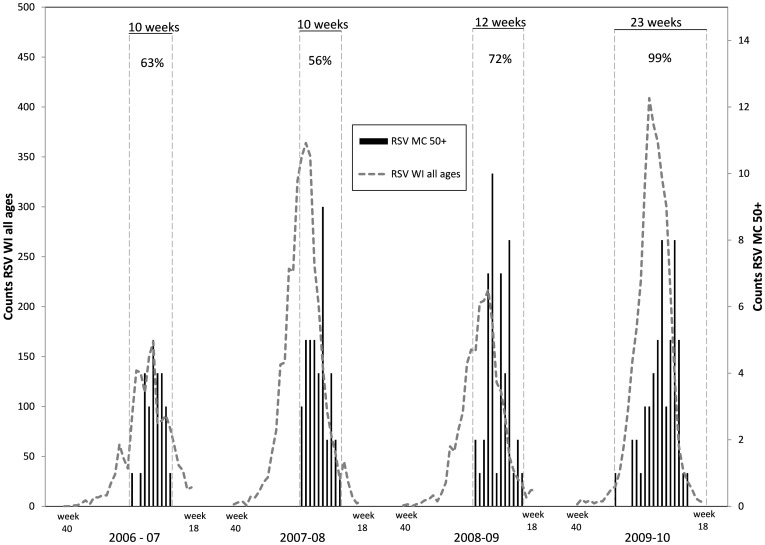
Medically attended RSV cases in Marshfield study participants ≥50 years old and statewide RSV counts (all ages) based on testing at the Wisconsin State Laboratory of Hygiene. Study enrollment periods are identified by vertical dashed lines; the percent of all statewide cases that occurred during each enrollment period is shown at the top.

**Figure 2:**
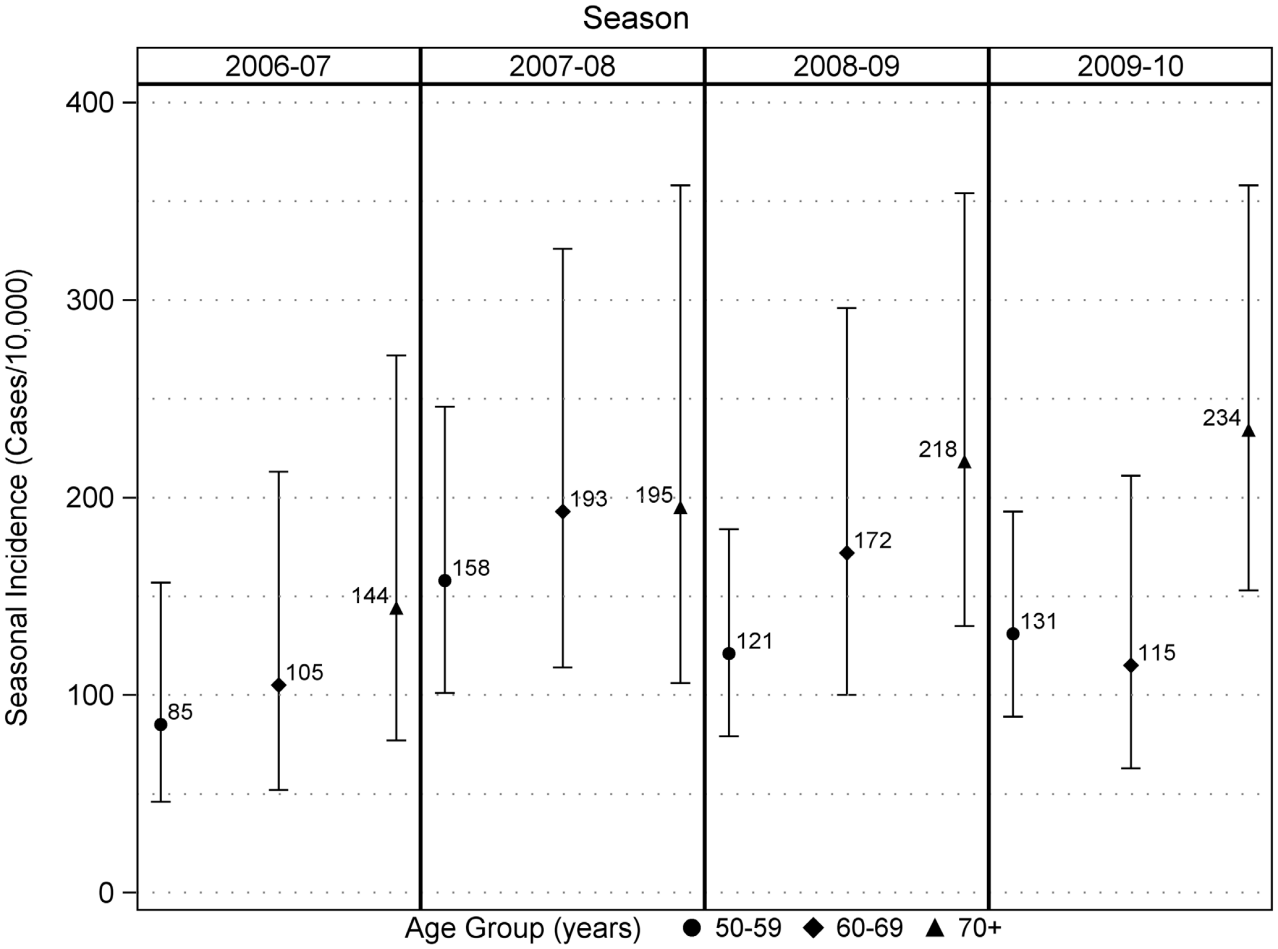
Seasonal incidence and 95% confidence limits of medically attended RSV by age group in a community cohort of adults >50 years old.

The enrollment period included the peak week of RSV activity in Wisconsin during every season, and the proportion of statewide cases that occurred during the enrollment period varied from 56% to 99% (Figure 1). Across all seasons, medically attended RSV incidence (cases per 10,000 persons per season) was 154 (95% CI, 132–180) (Table 3). The incidence was 178 (95% CI, 147–216) in females and 127 (95% CI, 99–163) in males (p = 0.03). Among persons 50–59, 60–69, and ≥70 years old, RSV incidence was 124 (95% CI, 99–156), 147 (95% CI, 110–196), and 199 (95% CI, 153–258), respectively. Medically attended RSV incidence increased with increasing age group (p = 0.048, chi-square test for trend). For persons of age 60 years and greater, the incidence was 176 (95% CI, 144–214). There was no consistent trend in RSV incidence over time during the four seasons that were included in this analysis (Table 2). It was lowest in 2006–07 season and highest in the 2007–08 season, but differences between seasons were modest (Figure 2).

**Table 3:** Estimated seasonal incidence per 10,000 persons and 95% confidence limits (CL) of medically attended RSV (total, A or B) in adults ≥50 years old by season, gender and age group, in a Marshfield Wisconsin community cohort, 2006–07 through 2009–10.

	Seasonal Incidence/10,000 (95% CL)		
	RSV	RSV A	RSV B
Overall	154 (132,180)	80 (64,100)	74 (59,93)
Season			
2006–07	110 (75,161)	69 (42,112)	41 (21,80)
2007–08	179 (132,244)	86 (53,138)	93 (61,143)
2008–09	166 (125,221)	33 (16,67)	133 (97,184)
2009–10	159 (122,208)	131 (97,177)	28 (15,54)
Gender			
Male	127 (99,163)	65 (46,93)	61 (42,89)
Female	178 (147,216)	93 (70,124)	85 (64,113)
Age Group			
50–59 years	124 (99,156)	59 (42,83)	66 (47,91)
60–69 years	147 (110,196)	87 (59,130)	59 (37,94)
≥70 years	199 (153,258)	102 (70,149)	97 (65,143)

The incidence of medically attended RSV A and RSV B was similar overall, and when stratified by age group and gender. However, there was seasonal variation in the dominant type. RSV B was dominant in 2008–09 and RSV A was dominant in 2009–10 (Table 3). During the 2006–07 and 2007–08 seasons, the incidence of RSV A and RSV B was similar.

## Discussion

This study provides evidence that RSV is a common cause of outpatient respiratory illness among community-dwelling adults ≥50 years old. The seasonal incidence of medically attended RSV ranged from 110 to 179 episodes per 10,000 individuals. It was highest in the oldest age group, and it was higher in females compared to males. These differences may be due to age and gender differences in health care seeking behavior for acute respiratory illness [21]. The RSV incidence was nearly identical in three of four seasons (2007–08 through 2009–10), a time period when seasonal influenza activity varied greatly [22]. There were inconsistent differences in the incidence of medically attended RSV A and RSV B across seasons. In the first two seasons, the incidence of RSV A and B were similar, but each of the last two seasons was strongly dominated by either type B (in 2008–09) or type A (in 2009–10).

In the Marshfield cohort, the seasonal incidence of medically attended RSV was similar to what was reported from a prospective cohort study of healthy elderly adults (≥65 years old) in Rochester, New York [7]. The Rochester study occurred over four consecutive winters from late 1999 to early 2003, and they conducted surveillance for all RSV infections rather than only medically attended RSV. The seasonal incidence of RSV infection was reported to be 300 to 700 per 10,000 elderly adults. In the Rochester study, 17% of RSV infections resulted in a health care encounter. If we apply this proportion to the total number of RSV cases in the healthy elderly Rochester cohort, we estimate that the seasonal incidence of medically attended RSV was approximately 50 to 120 episodes per 10,000. The Rochester study also followed a cohort of high risk adults with chronic heart or lung disease, and the estimated seasonal incidence of medically attended RSV was higher (approximately 180 to 440 per 10,000) in that group.

Although the incidence of medically attended RSV in Marshfield and Rochester was similar during different time periods, comparisons are limited by differences in the study design. Our study captured only cases of medically-attended RSV infection, and we were unable to identify cases of RSV that were managed at home. In contrast, the Rochester study conducted active surveillance in the cohort to identify all incident cases of RSV, including illnesses that did not lead to a health care encounter. The studies also differed in terms of age distribution and diagnostic testing methods. The Rochester cohort was older with a mean age of 75 years compared to 64 years in the Marshfield cohort. Although both studies used RT-PCR for virus detection, the Rochester study also obtained acute and convalescent sera from ill individuals. One third of RSV cases were identified based on seroconversion without RT-PCR confirmation.

In our study, six percent of patients with RT-PCR confirmed RSV illness were hospitalized within 14 days after illness onset. By applying this proportion to the numerator for our estimates of population incidence, we would estimate approximately 9 RSV hospitalizations per 10,000 adults ≥50 years old each season. However, the number of RSV hospitalizations was low, and we had insufficient power to evaluate the incidence of hospitalization. A hospital-based surveillance study in Davidson County, Tennessee from 2006–07 through 2008–09 reported that the average annual rate of RSV hospitalization was 15 per 10,000 residents [2].

Strengths of this study include systematic screening and enrollment from a well-defined community cohort, testing early in the course of illness with prospective follow-up to identify hospital admissions, and inclusion of multiple seasons. The study was limited by the need to make assumptions regarding the proportion of non-enrolled cohort members with RSV, and the number of RSV cases that occurred outside the enrollment window each season. The assumption regarding non-enrolled patients was supported by our observation that seven common diagnosis codes accounted for 82% to 90% of all codes given during acute respiratory illness visits for both enrolled and non-enrolled individuals with any acute respiratory illness. The assumption regarding the proportion of cases occurring outside each seasonal enrollment window was necessarily based on test results from the Wisconsin State Laboratory of Hygiene.

RSV infections can be particularly severe in adults with chronic disease [1], [7], but laboratory diagnosis of RSV remains uncommon. The results of this study identify RSV as a common cause of acute respiratory illness among community-dwelling older adults in the outpatient setting. The time lag we observed between the statewide RSV peak and the peak incidence in our population of older adults is consistent with a prior study in the United Kingdom that compared the incidence of lower respiratory tract infection in adults with the number of RSV positives based on clinical testing over 10 seasons [23]. In both Marshfield and the United Kingdom, there was a time lag of a few weeks between the peak RSV occurrence based on clinical testing (largely in children) and the peak incidence in older adults during most seasons. These findings suggest that children may be an important source of RSV transmission to older adults, and interventions to prevent RSV in children may also benefit the older adult population. Greater knowledge regarding the epidemiology and burden of RSV disease in older adults will be useful for future vaccine development and licensure.
